# Differences in cNOS/iNOS Activity during Resistance to Trypanosoma cruzi Infection in 5-Lipoxygenase Knockout Mice

**DOI:** 10.1155/2019/5091630

**Published:** 2019-10-24

**Authors:** Carolina Panis, Vanessa Jacob Victorino, Vera Lúcia Hideko Tatakihara, Rubens Cecchini, Luiz Vicente Rizzo, Lucy Megumi Yamauchi, Sueli Fumie Yamada-Ogatta, Marli Cardoso Martins-Pinge, Phileno Pinge-Filho

**Affiliations:** ^1^Laboratório de Mediadores Inflamatórios, Universidade Estadual do Oeste do Paraná, Francisco Beltrão, Paraná 85605-010, Brazil; ^2^Laboratório de Imunopatologia Experimental, Centro de Ciências Biológicas, Universidade Estadual de Londrina, Londrina, 86051-970 Paraná, Brazil; ^3^Laboratório de Patofisiologia e Radicais Livres, Centro de Ciências Biológicas, Universidade Estadual de Londrina, Londrina, Paraná 86051-970, Brazil; ^4^Hospital Israelita Albert Einstein, Avenida Albert Einstein 627-701, Subsolo Bloco A., 05651-901 São Paulo, São Paulo, Brazil; ^5^Departamento de Microbiologia, Centro de Ciências Biológicas, Universidade Estadual de Londrina, Londrina, Paraná 86051-970, Brazil; ^6^Departamento de Ciências Fisiológicas, Centro de Ciências Biológicas, Universidade Estadual de Londrina, Londrina, Paraná 86051-970, Brazil

## Abstract

Infection with the protozoan *Trypanosoma cruzi* causes Chagas disease and consequently leads to severe inflammatory heart condition; however, the mechanisms driving this inflammatory response have not been completely elucidated. Nitric oxide (NO) is a key mediator of parasite killing in *T. cruzi*-infected mice, and previous studies have suggested that leukotrienes (LTs) essentially regulate the NO activity in the heart. We used infected 5-lipoxygenase-deficient mice (5-LO^−/−^) to explore the participation of nitric oxide synthase isoforms, inducible (iNOS) and constitutive (cNOS), in heart injury, cytokine profile, and oxidative stress during the early stage of *T. cruzi* infection. Our evidence suggests that the cNOS of the host is involved in the resistance of 5-LO^−/−^ mice during *T. cruzi* infection. iNOS inhibition generated a remarkable increase in *T. cruzi* infection in the blood and heart of mice, whereas cNOS inhibition reduced cardiac parasitism (amastigote nests). Furthermore, this inhibition associates with a higher IFN-*γ* production and lower lipid peroxidation status. These data provide a better understanding about the influence of NO-interfering therapies for the inflammatory response toward *T. cruzi* infection.

## 1. Introduction

Infection with the hemoflagellate *Trypanosoma cruzi* causes Chagas disease, essentially leading to morbidity and mortality in Latin America [1–3]. The parasite hampers the host immune response during the acute infection phase, thereby leading to chronic phase with distinct clinical evolution [4]. Early immune response against *T. cruzi* depends on several signaling factors, such as the production of Th1/Th2 cytokines and chemokines [5, 6], eicosanoids [7], and nitric oxide (NO) [8]. NO is crucial in determining the disease outcome against *T. cruzi* infection [9].

In general, NO is produced mainly from nitric oxide synthase (NOS) activities, presented as inducible (iNOS/NOS2) or constitutive isoforms (cNOS). cNOS are calcium-dependent and include neuronal NOS (NOS1) as well as endothelial NOS (NOS3). iNOS is regulated by several factors such as cytokines and microbial-derived products yielding abundant NO [10], whereas cNOS is physiologically expressed, generating low levels of NO [11].

Evidence implicates that NO is pivotal in controlling the parasite burden in experimental *T. cruzi* infection [12–14], mostly related to the overexpression or enhanced activity of iNOS. In particular, iNOS activation, proinflammatory cytokines, and chemokines produced by cardiomyocytes presumably control the parasite growth and cell influx, thus contributing to the pathogenesis of Chagasic cardiomyopathy as observed in *T. cruzi*-infected mice [15–17] and rhesus monkeys [14], in addition to Lewis rats infected with *T. cruzi* (Sylvio X10/7 strain) [18].

NO in the heart is derived from the three NOS isoforms [19]. An advanced study using a mouse model of *T. cruzi* infection demonstrated that NO can be regarded as a “double-edged” sword [20]. Despite the importance of NO derived from iNOS to the intracellular killing of parasites, it may lead to myocardial dysfunction [17]. Another study considered iNOS as inessential in controlling *T. cruzi* infection [21], suggesting the implication of other additional mechanisms in parasite control. In this scenario, other regulatory factors against *T. cruzi* have emerged, such as the eicosanoids [7, 22, 23].

Leukotrienes (LTs) enhanced the ability of macrophages in eliminating *T. cruzi* infection [24] and may develop resistance to any infection in a NO-dependent manner [25–28]; in addition, LT deficiency impairs the host immunity against *T. cruzi* [26, 27]. These results show 5-lipoxygenase (5-LO) as an important pathway during NO production due to *T. cruzi* infection.

In this context, we used 5-LO^−/−^ deficient mice to investigate the participation of iNOS/cNOS in the heart, oxidative stress, and cytokine profile during the acute infection phase. We found that the cNOS appears to act via mechanisms that favor the parasite survival, whereas the iNOS modulates the infection by maximizing the trypanocidal mechanisms of the host. Thus, this study is the first to demonstrate the differences in the cNOS/iNOS activity considering the resistance toward *T. cruzi* infection in 5-LO^−/−^ mice.

## 2. Materials and Methods

### 2.1. Animals

Mice (6-10 weeks old, 20–30 g) with a targeted disruption of the 5-LO gene (5-LO^−/−^) [29] and littermate wild-type (WT) controls (129 WT) were purchased from the Jackson Laboratories and were kindly provided by Dr. Fernando Queiroz Cunha (University of Sao Paulo, Ribeirao Preto, Brazil). The animals were housed in a controlled environment and were provided with standard rodent chow and water.

This study was carried out in strict accordance with the principles and guidelines adopted by the Brazilian National Council for the Control of Animal Experimentation (CONCEA), and the technical procedures were approved by the Ethical Committee on Animal Use (CEUA), State University of Londrina (CEUA/UEL: protocol 28568). All surgical procedures were performed under ketamine/xylazine hydrochloride anesthesia, and care was taken to minimize animal suffering.

### 2.2. Parasite and Infection

*T. cruzi* (Y strain) [30], belonging to the TcI lineage [31], was kindly provided by Dr. Paulo Araújo, Campinas State University, Brazil, and was maintained by weekly intraperitoneal (i.p.) inoculation of 2 × 10^5^ trypomastigote forms on Swiss mice. For experiments, blood was obtained by cardiac puncture with heparinized syringes and needles. Trypomastigote forms were enumerated in a hematocytometer, and 5 × 10^3^ forms were injected i.p. in mice. Aminoguanidine (AG, selective iNOS inhibitor) [32] and nonspecific NOS inhibitor L-nitroarginine methyl ester (L-NAME) [33] (Sigma-Aldrich, St. Louis, MO, USA) were diluted in sterile phosphate buffer saline (PBS pH 7.2). All solutions were freshly prepared, under sterile conditions.

### 2.3. Treatment of Animals

The mice received daily i.p. injections of aminoguanidine (AG, 50 mg/kg/day), L-NAME (LN, 20 mg/kg/day), or injections containing both inhibitors in the aforementioned concentrations. The first dose was administered at 4 h after *T. cruzi* infection. The inhibitor dose selected for these experiments was based on the previously published studies demonstrating its efficacy [34–36]. Control experimental groups (*n* = 3‐5 animals) received PBS (0.2 mL) by the same route.

### 2.4. Parasitemia and Survival Rates

Parasitemia was accessed under standardized conditions, by direct microscopic observation of 50 fields in 5 *μ*L of heparinized tail venous blood on alternate days from the 5th day after *T. cruzi* infection. Data were expressed as number of parasites/per microliter [37]. Survival rate of the infected groups was evaluated for 30 days.

### 2.5. Blood Collection and Determination of Nitric Oxide (NO) Levels

The control (noninfected) and infected animals (day 12 after infection) were anesthetized, and heparinized blood was collected by cardiac puncture. The plasma was obtained by blood centrifugation at 600 *g*, for 10 min, at 4°C and was stored at −20°C until further analysis. Plasmatic NO was measured as nitrite by reducing nitrate to nitrite with cadmium granules, followed by Griess reaction, as we previously described [26, 38]. Nitrite concentration was quantified using various NaNO_2_ concentrations as standard, and data were expressed in micromoles.

### 2.6. Cytokine and Eicosanoid Production

Plasma cytokine levels (IL-2, IL-4, IL-5, IL-6, IL-10, IL-12, IL-13, 1L-17A, IL-23, IFN-*γ*, TNF-*α*, and TGF-*β*1) were determined using a Multi-Analyte Profiler ELISArray® (Superarray Bioscience Corporation, CAT#1026 A). Eicosanoid levels were assessed employing the acetylcholinesterase-based assay PGE_2_ EIA kit and leukotriene B_4_ EIA kit (Cayman Chemical, CAT#514531).

### 2.7. Cardiac Parasitism

The mouse hearts were removed on day 12 i.p., sliced transversally in three sections, and were fixed in 10% buffered formalin. Paraffin-embedded 5 mm sections were stained with hematoxylin-eosin stain. Tissue parasitism was evaluated by enumerating the amastigote nest count visualized in three sections per animal [39].

### 2.8. Chemiluminescence Assay

To estimate plasmatic lipoprotein peroxidation, 125 *μ*L of plasma was diluted in 865 *μ*L phosphate buffer (K_2_HPO_4_ 30 mM in 1.15% KCL, pH = 7.4, 37°C) and 10 *μ*L of tert-butyl hydroperoxide was added. Thereafter, the microtube was quickly put inside a Glomax 20/20 luminometer. Each sample was analyzed for 60 minutes, and the results were achieved from the peaks and the initiation time of the obtained curve [40].

### 2.9. Total Antioxidant Capacity of Plasma (TRAP)

TRAP was determined using 2,2′-azobis (ABAP) as a radical generator and luminol to amplify photon detection and light emission using chemiluminescence (CL), according to the method of Repetto et al. Plasma aliquots were diluted at 1 : 50 ratio, and 70 *μ*L of these aliquots was added to 830 *μ*L of glycine buffer of 0.1 M pH 8.6 and 50 *μ*L of luminol solution (3.98 mg in 250 *μ*L of KOH 1 M). About 50 *μ*L of 2,2′-azobis (ABAP) solution (54.24 mg/mL) was added to initiate the reaction. ABAP reacts with lipids present in the plasma, thus forming lipoperoxides that emit photons in the presence of luminol; however, this reaction is inhibited by low molecular antioxidant substances in the plasma [40, 41]. CL curves were obtained in a GloMax luminometer (TD 20/20, Turner Designs), and the results are expressed in nM of Trolox.

### 2.10. Statistical Analysis

Data were processed and analyzed by the analysis of variance (ANOVA) and the Bonferroni posttest or unpaired *t*-tests using GraphPad Prism 6.0 software (La Jolla, CA, USA). Two-tailed values with 95% confidence intervals were acquired. Data are represented as the error of the mean (SEM). Values of *p* < 0.05 were considered as statistically significant.

## 3. Results

### 3.1. 5-LO Participates in Parasite Burden Control during T. cruzi Infection

**Figure 1:**
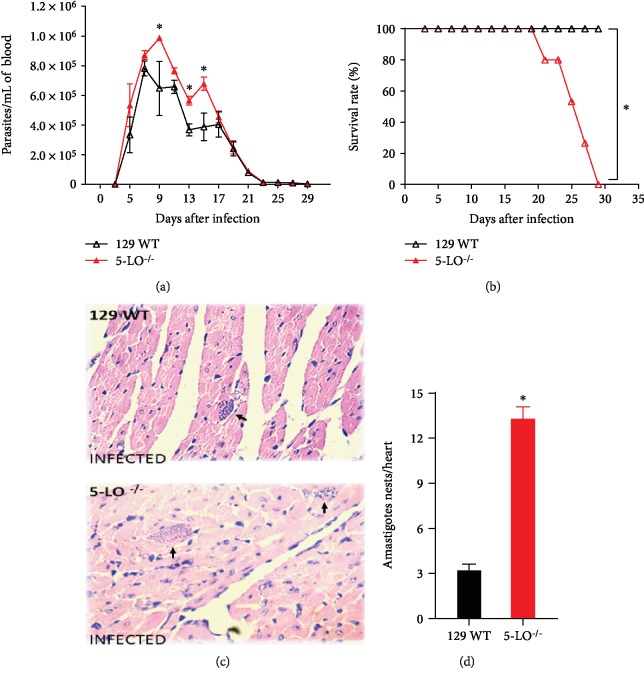
5-LO pathway signaling is essential for mouse resistance and control of parasitism in *T. cruzi* infection. (a) Parasitemia and (b) survival rate of 129 WT and 5-LO^−/−^ mice infected with 5 × 10^3^ trypomastigote forms of *T. cruzi* Y strain. (c) Representative images from hematoxylin-eosin staining of heart tissue from 129 WT and 5-LO^−/−^ mice 12 days of postinfection. Black arrows indicate amastigote nests. (d) Cardiac parasitism of three heart sections enumerated per mouse. *N* = 3‐5 animals in each group and values are the mean ± SEM. ^∗^*p* < 0.001.

Wild-type (129 WT) and 5-LO^−/−^ mice were infected with *T. cruzi*; parasitemia and survival were monitored according to Panis et al. [26]. Data revealed that the infection peak occurred around the 7th day for both strains (Figure 1(a)). 5-LO^−/−^ presented higher parasitemia levels and lower circulating parasites from the 15th day of infection (Figure 1(a)). Regarding mouse survival rates, we observed that all 129 WT animals infected survived until the 30th day of infection, whereas all the knockout animals died within this period (Figure 1(b)). These data confirmed that the 5-LO signaling pathway is crucial for the animals' survival and resistance to *T. cruzi* infection, as previously described [26]. Moreover, cardiac parasitism (postinfection, 12th day) in knockout mice revealed that the amastigote nests were four times higher than those in 129 WT mice (13.66 ± 1.56 vs. 3.32 ± 0.51, respectively; Figures 1(c), 1(d)), suggesting that the control of multiplication of the parasite in the heart tissue is associated with the 5-LO presence.

### 3.2. Contrasting Effects of iNOS and cNOS Blockage on Parasite Loading and Relation with Leukotriene Lacking

**Figure 2:**
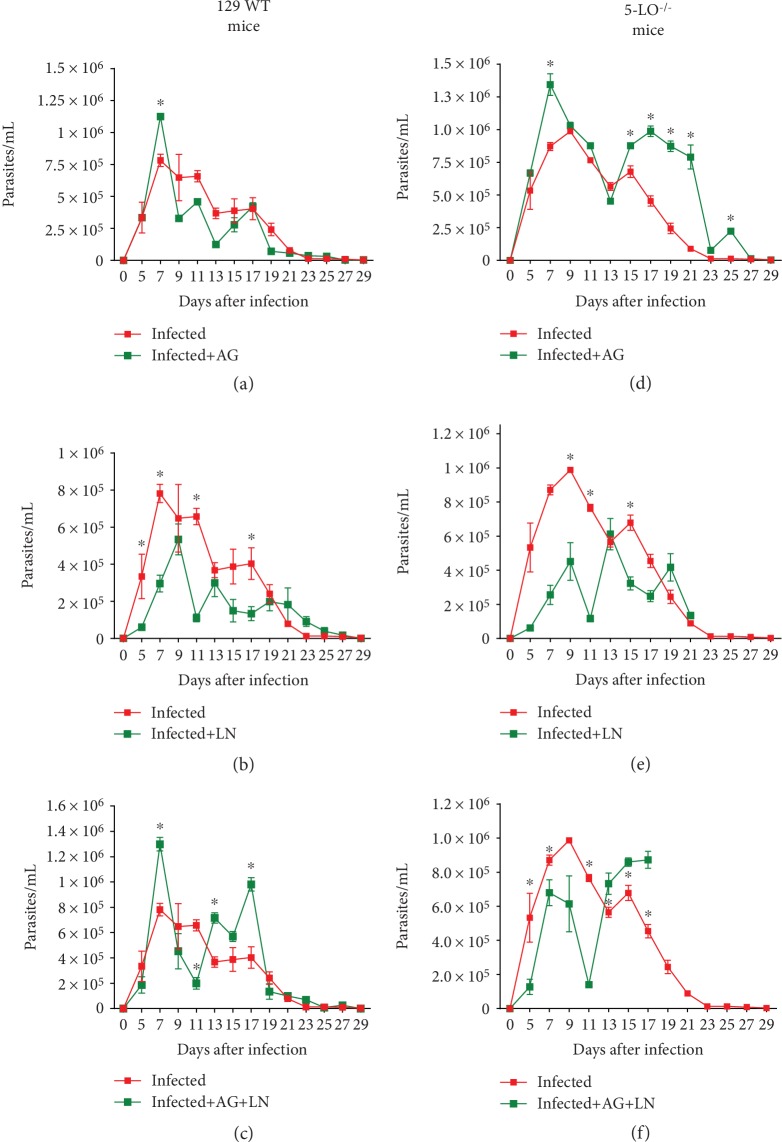
Differences in cNOS/iNOs activity considering the resistance to *T. cruzi* infection. (a–f) Parasitemia of 129 WT and 5-LO^−/−^ mice infected with 5 × 10^3^ trypomastigote forms of *T. cruzi* Y strain. Mice were treated 4 h before infection and were administered i.p. daily for 30 days, employing inhibitors dose as follows: aminoguanidine (AG, iNOS inhibitor, 50 mg/kg/day), N*ω*-nitro-L-arginine methyl ester hydrochloride (L-NAME, 20 mg/kg/day), or both. Control experimental groups received PBS (0.2 mL/mouse). *N* = 5 animals in each group and values are the mean ± SEM. ^∗^*p* < 0.001.

**Figure 3:**
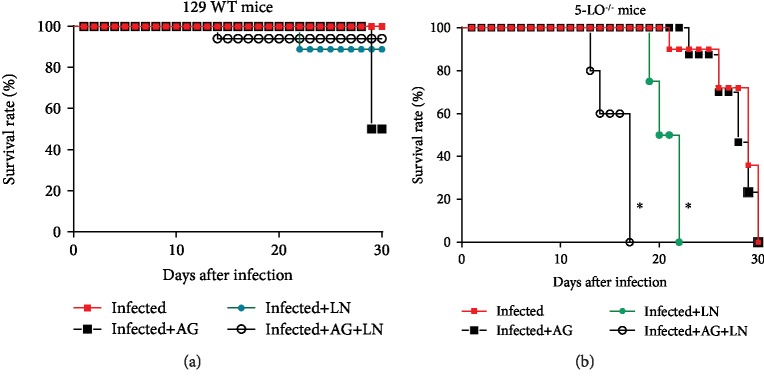
Survival rate of *T. cruzi*-infected mice treated with iNOS/cNOS blockers. (a) 129 WT mice and (b) 5-LO^−/−^ mice infected with 5 × 10^3^ trypomastigote forms of *T. cruzi* Y strain. Mice were treated 4 h before infection and were administered i.p. daily for 30 days, employing inhibitors dose as follows: aminoguanidine (AG, iNOS inhibitor; 50 mg/kg/day), N*ω*-nitro-L-arginine methyl ester hydrochloride (L-NAME [57], 20 mg/kg/day), or both. Control experimental groups received PBS (0.2 mL/mouse). *N* = 5 animals in each group and values are the mean ± SEM. ^∗^*p* < 0.001.

**Figure 4:**
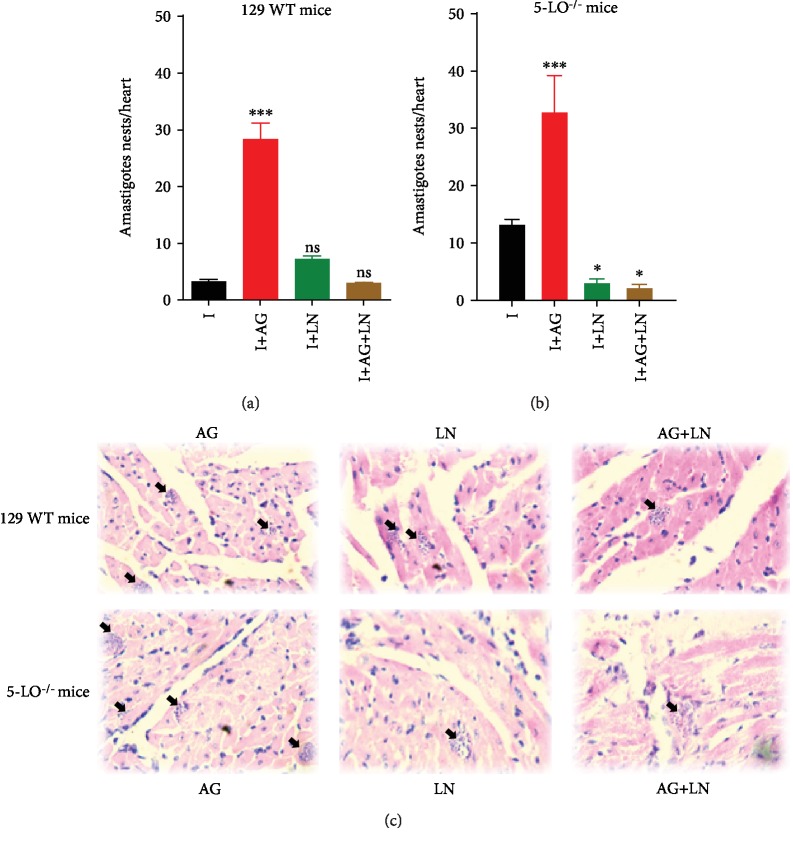
Cardiac parasitism. Number of amastigote nests in (a) wild-type (129 WT mice) and (b) 5-LO^−/−^ mice infected with 5 × 10^3^ trypomastigote forms of *T. cruzi* (day 12 of postinfection). (c) Photomicrographs of cardiac sections stained with hematoxylin-eosin. AG: infected mice treated with aminoguanidine (50 mg/kg/day i.p.); LN: infected mice treated with L-NAME (20 mg/kg/day i.p.); AG+LN: infected mice treated with aminoguanidine (50 mg/kg/day i.p. plus L-NAME 20 mg/kg/day). *n* = 3 animals in each group and values are the mean ± SEM for three independent experiments, ^∗^*p* < 0.001. ns: not significant, *p* > 0.05 compared with control group (untreated and infected).

Considering the confirmation that our disease model repeated the results previously obtained [26] as well as the existing relation between LTs and NO [25], we assessed the role of iNOs and cNOs enzymes during the acute phase of *T. cruzi* infection. The administration of aminoguanidine (AG) in our data corroborates the findings of previous studies [12], by demonstrating the involvement of iNOS during the acute *T. cruzi* infection. We found a higher number of parasites in the animals treated with AG for both strains (Figures 2(a), 2(d))—with 50% of mortality of 129 WT mice until the 28th day after the infection (Figure 3(a)) and 100% of the knockout group until the 30th day (Figure 3(b)). The assessment of the amastigote nest count in the cardiac tissue (Figure 4) for the group treated with AG revealed higher cardiac parasitism in both strains in a similar manner (129 WT = 28.33 ± 4.33; knockout = 33.01 ± 10.81).

To determine the role of cNOS in acute infection, we subjected the animals to a 30-day L-NAME (LN) injection. We found that the blockage decreased the number of parasites in both strains (Figures 2(b), 2(e)), consequently promoting lower parasite levels in the knockout group (9th day after infection, Figures 2(b), 2(e)). Survival rates in 129 WT animals treated with L-NAME promoted 10% of mortality after 30 days (Figure 3(a)) and 100% of mortality for the knockout mice within 22 days (Figure 3(b)). Figures 4(b), 4(c) illustrate the blockade of cNOS providing control of heart parasitism in the knockout animals without changing the nest count in WT animals (Figures 4(a), 4(c)).

In order to assess the simultaneous contribution of iNOS and cNOS in infection control, the mouse groups received concurrent injections of AG and L-NAME. Treatment with AG+L-NAME (AG+LN) enhanced the parasite burden only in the WT group, including a new peak at the 17th day (Figure 2(c)), and reduced the number of blood parasites in the knockout strain until the 11th day as well as increased parasite load from the 13th day (Figure 2(f)). The effect of this simultaneous blockade on the survival rate of WT mice did not cause significant survival reduction (Figure 3(a)), whereas the knockout strain revealed 100% mortality at the 17th day (Figure 3(b)). Interestingly, no modification on the cardiac parasite load occurred in WT mice with the simultaneous blockage of iNOS and cNOS, regardless of the presence of leukotrienes (Figures 4(a), 4(c)). Notably, the treatment with AG+L-NAME (AG+LN) provided the knockout animals with heart parasitism control (Figures 4(b), 4(c)).

### 3.3. NO Production during Acute Infection by T. cruzi Depends on Both iNOS and cNOS, Occurring in the Absence of Leukotrienes

Even though production of iNOS-derived NO during acute *T. cruzi* infection is considered indispensable for host survival, the role of cNOS-derived NO during infection remains unclear. Considering that LTs and NO are important mediators for host survival against *T. cruzi* [25, 26], we investigated the effects of cNOS and iNOS blockage in defining the key source enzyme of NO in the presence and absence of 5-LO.

**Figure 5:**
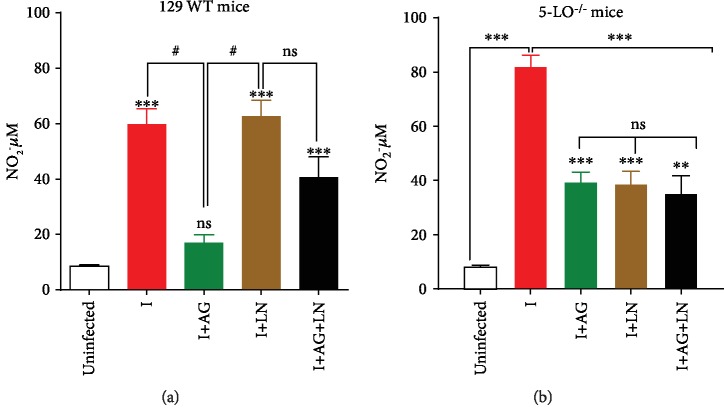
Nitric oxide (NO) production. (a) Wild-type (129 WT mice) and (b) 5-LO^−/−^ mice infected with 5 × 10^3^ trypomastigote forms of *T. cruzi* Y strain and treated or untreated with NOS inhibitors. NO was determined by measuring the nitrite levels in plasma employing the cadmium-copper system followed by the Griess reaction. *N* = 5 animals in each group and values are the mean ± SEM for three independent experiments. ^∗^*p* < 0.001. ^#^*p* < 0.001, ns: not significant, *p* > 0.05 compared with uninfected group or between groups.

Our findings [25, 42] revealed the occurrence of a basal NO production in control animals (uninfected), regardless of the presence of LTs (Figures 5(a), 5(b)). The infection increased the NO levels in 129 WT animals (8.44 ± 1.47 to 59.781 ± 13.69, Figure 5(a)) as well as in knockout mice (from 8.24 ± 1.39 to 82.04 ± 8.45, Figure 5(b)) indicating a higher NO production during infection upon the absence of LTs.

Treatment with AG generated lower NO levels in infected 129 WT mice (from 59.78 ± 13.69 to 16.96 ± 6.54, Figure 5(a)) and infected 5-LO^−/−^ mice (from 82.04 ± 8.45 to 39.56 (Figure 5(b)). cNOS inhibition led to a significant decrease in NO production only in the infected knockout animals (82.04 ± 8.45 to 38.79 ± 16.9; Figure 5(b)), whereas the coinhibition of iNOS and cNOS caused a significant decrease only in the 5-LO^−/−^ group (from 82.04 ± 8.45 to 35.17 ± 14; Figure 5(b)).

### 3.4. Cytokine Production Is Influenced by Leukotrienes and Altered by NOS Blockage

**Figure 6:**
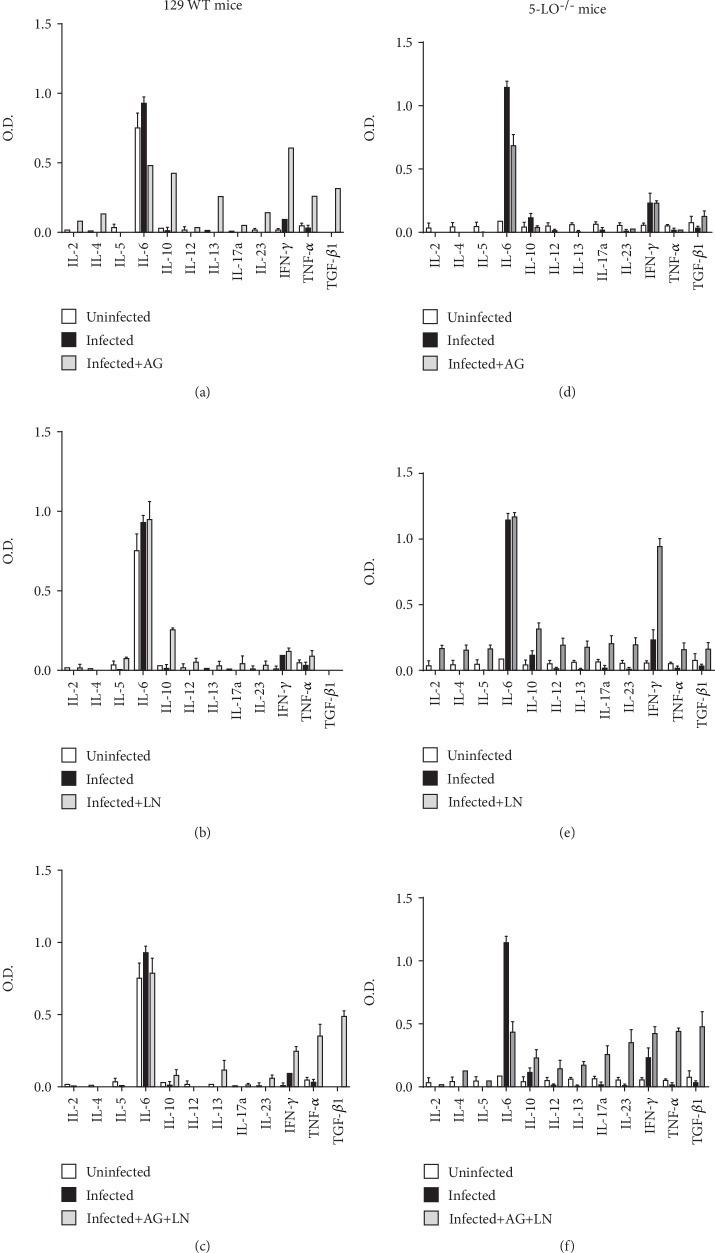
Plasma levels of cytokines after treatment with NOS inhibitors. Three independent samples, each for 129 WT mice (a–c) and 5-LO^−/−^ mice (d–f), were used. AG: infected mice treated with aminoguanidine (50 mg/kg/day i.p.); LN: infected mice treated with L-NAME (LN; 20 mg/kg/day i.p.); AG+LN: infected mice treated with aminoguanidine (50 mg/kg/day i.p. plus L-NAME 20 mg/kg/day). Mean OD values of Multi-Analyte ELISArray results for indicated cytokines are presented; OD > 1.0 is indicated as 1.0. Data are representative of two independent experiments. *N* = 5 animals in each group and values are the mean ± SEM. ^∗^*p* < 0.001.

In order to elucidate whether the production of cytokines is influenced by leukotrienes and the blockage of iNOS and cNOs during *T. cruzi* infection, we established the levels of Th1/Th2 systemic cytokines in both mouse strains (Figure 6). Basal levels found in the control groups (uninfected) reveal that the knockout mice displayed lower levels of IL-6 than the WT (Figure 6(d)), in addition to low levels of IL-13, IL-17a, IL-23, and TGF-*β*1 (Figures 6(a), 6(d)). Notably, we observed an increment in IL-6 production during acute *T. cruzi* infection for both 129 WT and knockout mice (Figures 6(a), 6(d)).

iNOS blockage generated higher IL-10, IL-13, IFN-*γ*, TNF-*α*, and TGF-*β*1 in WT mice along with a lower IL-6 reduction in relation to untreated *T. cruzi* animals (Figure 6(a)). Knockout animals revealed lower IL-6 levels during aminoguanidine (AG) treatment in relation to the infected untreated mice (Figure 6(d)). cNOS inhibition promoted an increment in IL-10 production in 129 WT and 5-LO^−/−^ mice compared with the untreated mice (Figures 6(b), 6(e)) and had higher IFN-*γ* in 5-LO^−/−^ mice (Figure 6(e)). Coinhibition increased the levels of IFN-*γ*, TNF-*α*, and TGF-*β*1 in the 129 WT group (Figure 6(c)), whereas the knockout group revealed lower IL-6 in addition to augment the IL-10, IL-12, IL-13, IL-17a, IL-23, IFN-*γ*, TNF-*α*, and TGF-*β*1 levels (Figure 6(f)).

### 3.5. NOS Blockage Alters Oxidative Stress Levels during T. cruzi Infection

Considering that lipoxygenase pathway is crucial in erythrocyte oxidative stress through NO in Chagas' disease [27], we analyzed the effect of iNOS and cNOs blockage on the antioxidant/oxidative status during early *T. cruzi* infection.

**Figure 7:**
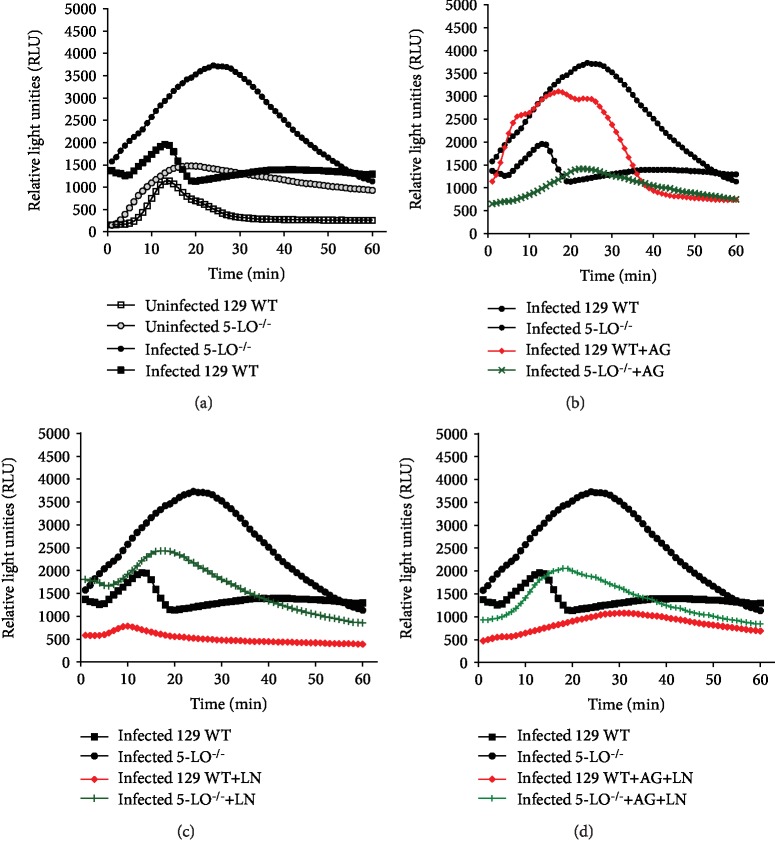
Oxidative stress profile evaluated by tert-butyl hydroperoxide-initiated chemiluminescence on day 12 after *T. cruzi* infection. (a–d) Mice were infected with 5 × 10^3^ trypomastigote forms of *T. cruzi* (Y strain) and were treated or untreated with NOS inhibitors. (a) Uninfected mice and untreated *T. cruzi*-infected mice were used as controls. (b) AG: infected mice treated with aminoguanidine (50 mg/kg/day i.p.); (c) LN: infected mice treated with L-NAME (LN; 20 mg/kg/day i.p.); (d) AG+LN: infected mice treated with aminoguanidine (50 mg/kg/day i.p. plus L-NAME 20 mg/kg/day). *N* = 5 animals in each group. Values represent the mean ± SEM and are representative of two independent experiments ^∗^*p* < 0.001.

**Figure 8:**
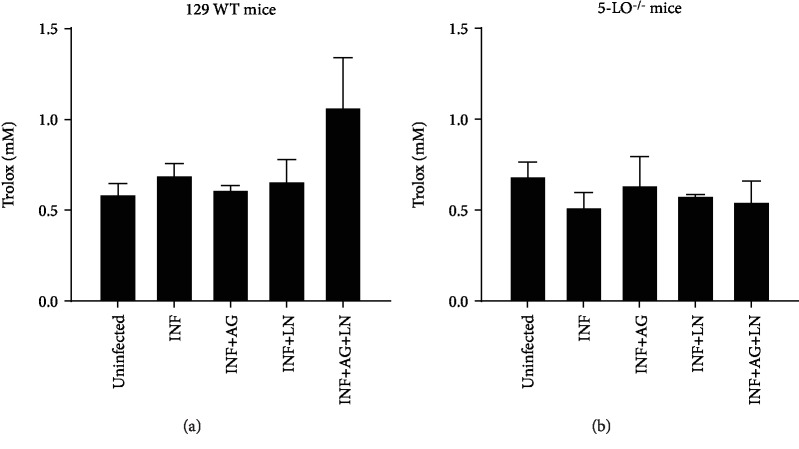
Plasmatic antioxidant profile. (a) Wild-type (129 WT mice) and (b) 5-LO^−/−^ mice. Mice were infected with 5 × 10^3^ trypomastigote forms of *T. cruzi* (Y strain) and were treated or untreated with NOS inhibitors. AG: infected mice treated with aminoguanidine (50 mg/kg/day i.p.); LN: infected mice treated with L-NAME (LN; 20 mg/kg/day i.p.); AG+LN: infected mice treated with aminoguanidine (50 mg/kg/day i.p. plus L-NAME 20 mg/kg/day). Uninfected mice were used as controls. *N* = 5 animals in each group. Values represent the mean ± SEM and are representative of two independent experiments ^∗^*p* < 0.001.

According to Figure 7(a), the plasma of knockout mice presented higher basal levels of lipoperoxides than that of the 129 WT animals (Figure 7(a)). The infection led to increased levels of lipoperoxidation in both mouse strains, whereas 5-LO^−/−^ revealed higher levels compared to the 129 WT mice (Figure 7(a)). The AG administration generated higher stress levels in 129 WT infected mice as well as lower lipoperoxidation in infected knockout mice (Figure 7(b)). Additionally, L-NAME (LN) and the cotreatment with AG and L-NAME (LN) reduced lipoperoxidation in both mouse strains (Figures 7(c), 7(d)). TRAP levels did not reveal significant alterations (Figures 8(a), 8(b)).

### 3.6. Eicosanoid Balance during T. cruzi Infection and NOS Inhibition

**Figure 9:**
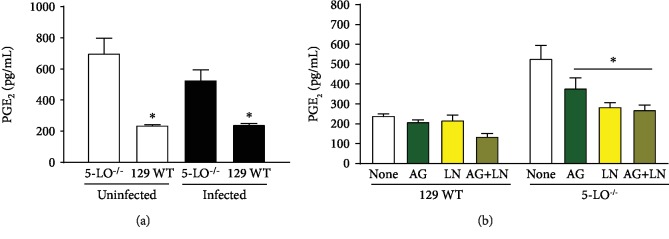
Plasma levels of PGE_2_. (a) Wild-type (129 WT mice) and 5-LO^−/−^ mice uninfected and infected and (b) treated or untreated with NOS inhibitors. AG: infected mice treated with aminoguanidine (50 mg/kg/day i.p.); LN: infected mice treated with L-NAME (LN; 20 mg/kg/day i.p.); AG+LN: infected mice treated with aminoguanidine (50 mg/kg/day i.p. plus L-NAME 20 mg/kg/day). Mice were infected with 5 × 10^3^ trypomastigote forms of *T. cruzi* (Y strain). *N* = 3 animals in each group and values are the mean ± SEM. ^∗^*p* < 0.001 compared with the control group.

We did not detect the production of LTB_4_ in the 5-LO^−/−^ plasma of the mice used in the study, regardless of the infection or treatment using NOS inhibitors (data not shown). Interestingly, the PGE_2_ amount released from 5-LO^−/−^ before or after *T. cruzi* infection was greater than that released from the wild-type plasma (Figure 9(a)). The treatment using NOS blockers generated lower PGE_2_ levels in the plasma only for the 5-LO^−/−^ infected mice (Figure 9(b)).

## 4. Discussion

Higher levels of iNOS expression have been associated with Chagas disease [16] since nitric oxide (NO) is toxic to *T. cruzi* [12, 43]; however, data from iNOS-deficient mice are controversial in the context of *T. cruzi* infection as the iNOS-deficient mice are resistant to *T. cruzi* [21]. Our study investigated the involvement of iNOS/cNOS during the acute *T. cruzi* infection by using 5-LO^−/−^ mice as an infection model.

Previous studies have reported that leukotrienes (LTs) are essential in NO production during parasitic infections, particularly those occurring due to from iNOS activity [25, 43–45]. Accordingly, infected 5-LO^−/−^ mice displayed lower NO production and iNOS protein expression in the heart compared to the WT infected mice, thus revealing that LTs may be required for optimum NO-dependent restriction of tissue parasitism [24]. In addition, different results for NO and cytokine productions occurred during the acute phase of *T. cruzi* infection in 5-LO^−/−^ mice, which has been related to resistance [28, 46] or susceptibility [26] presumably due to different trypomastigotes used to infect the experimental groups of mice.

Talvani et al. investigated the ability of LTB_4_ to induce NO production by macrophages infected with *T. cruzi* in vitro and whether NO mediated LTB4-induced parasite killing [25]. The authors demonstrated that the activation of macrophages with LTB_4_ induced a concentration- and time-dependent increase in NO production by *T. cruzi*-infected macrophages. Blockade of NO production with an iNOS inhibitor reduced the microbicidal activity of LTB_4_. In general, these results provide strong experimental evidence suggesting that the activation of *T. cruzi*-infected macrophages with LTB_4_ induces the production of TNF-*α*, which drives NO release. The NO produced was then responsible for parasite replication control [25].

We decided to apply an interventional strategy (5-LO gene deletion) [29] targeting both cysteinyl-LTs and LTB4. In particular, LTB_4_ production was not detected from the plasma of 5-LO^−/−^ mice used regardless of infection or treatment with NOS inhibitors. Unexpectedly, we present that the induction of elevated NO production in the plasma during *T. cruzi* acute infection occurred in the absence of 5-LO activity, suggesting that LT-independent mechanisms may be operative. The possible involvement of cNOS-derived NO against *T. cruzi* and its relationship with eicosanoids is yet to be investigated.

Our results revealed that iNOS blockage increased the circulating parasite load and reduced the overall survival in both mouse lineages in relation to the respective untreated infected groups; however, such effect was more prominent in the knockout group suggesting that iNOS is necessary to control the parasite load in the absence of 5-LO. Conversely, cNOS blockage significantly reduced the overall survival of 5-LO^−/−^ animals indicating that cNOS may modulate the parasite survival positively during acute *T. cruzi* infection.

During the *T. cruzi* infection, cardiac cells are extensively infected, and the amastigotes form several parasite nests. Cardiomyocytes are the cells that are able to express iNOS during *T. cruzi* infection and minimize its multiplication via this pivotal route [18, 47] presumably contributing to NO production in macrophages.

Accordingly, we demonstrated that 5-LO^−/−^ mice led to a fourfold increase process in amastigote nests in relation to the wild-type mice and revealed that iNOS is relevant for both the experimental groups used. Chandrasekar et al. [18] reported enhanced cardiac cNOS expression at the 3rd day after *T. cruzi* infection in rats, without any association with systemic NO production. Our results suggest the essential activation of the cNOS pathway in the absence of 5-LO metabolites that benefit parasite multiplication since the blockage of this system causes extremely low parasite loads compared to the untreated infected mice.

Measurement of plasma NO clearly presented the distinct involvement of NOS isoforms in acute infection: iNOS, but not cNOS, was essential for infected mice from the wild-type group since cNOS blockage revealed no alteration in the NO levels. In contrast, we found that cNOS blockage reduced the NO to the same levels of iNOS inhibition in the knockout group, which indicates that in the absence of LTs, cNOS-derivated NO must be an additional defense mechanism against *T. cruzi*.

Although 5-LO deficiency is a presumable limiting factor for the inflammatory response generation in the knockout animals, the production of proinflammatory cytokines and NO during *T. cruzi* infection has been described for this experimental model [26].

We demonstrated that the two strains of mice examined could respond to the parasite presence by producing cytokines despite distinct profiles. Knockout uninfected animals had lower basal levels of IL-6 compared to the wild-type mice, which may be related to their disability in the production of LTs, and consequently, low capacity to generate a satisfactory inflammatory response by T lymphocytes [48].

An increase in the proinflammatory cytokine IL-6 occurred during the acute infection phase with *T. cruzi* for both the wild-type and knockout mice. Low levels of IL-13, IL-17a, IL-23, and TGF-*β*1 were also found in these animals, suggesting that this mechanism can be a limiting factor to generate inflammatory response in the absence of leukotrienes. The higher levels of IL-6 verified may be associated with the role of this cytokine in the acute inflammatory response to infection through the activation and differentiation of T lymphocytes, stimulation of hepatic production of acute phase proteins, and antibody production by B lymphocytes [49].

We further observed that iNOS blockage increased the IL-10, IL-13, IFN-*γ*, TNF-*α*, and TGF-*β*1 in the WT mice along with IL-6 reduction compared to the untreated *T. cruzi*-infected mice. The cytokine profile observed in wild-type animals during iNOS blockage suggests a shift in the pattern of cellular response with the production of Th2 pattern cytokines. IFN-*γ* and TNF-*α* mediators are essential in NO production and antiparasitic responses [43]. Therefore, elevated levels may reflect a higher production of these cytokines to stimulate macrophages in order to increase the NO levels observed during iNOS blockade. Inhibition of cNOS during infection caused higher IL-10 production in the wild-type group and increased IFN-*γ* levels in knockout mice suggesting a change in cytokine profile upon cNOS absence for both mouse groups. Such alteration in the cytokine profiles did not modify the remaining parameters assessed during the course of infection in the WT group (cardiac parasitism, survival, parasitemia, and NO levels) indicating that cNOS does not participate in the regulatory mechanisms of protecting these animals in response to *T. cruzi* acute infection.

The involvement of cNOS during acute infection became quite pronounced in the knockout animals since its blockage induced higher plasma levels of IFN-*γ*, described as the main inducer of NO production [50]; however, such IFN-*γ* increase was not sufficient to maintain or increase the levels of NO produced by iNOS in response to infection during cNOS blockade in our model.

Blocking of NO production by cNOS and iNOS simultaneously generated higher levels of IFN-*γ*, TNF-*α*, and TGF-*β* in both strains demonstrating that these enzymes participate in the systemic processes regulating the patterns of cytokines produced in response to *T. cruzi*.

Peripheral polymorphonuclear cells are presumably the cellular types involved in NO production in the plasma. These results suggest that the endogenous production of lipid mediators and action on their respective receptors could be responsible for part of the IFN-*γ* ability to induce NO and TNF-*α* production in infected mice. In particular, an increment of IFN-*γ* occurred during the acute infection phase with *T. cruzi* infection in the knockout mice used (particularly those treated with NO inhibitors).

Despite the record of an alteration in the in vitro production of multiple cytokines by 5-LO-derived leukotrienes [51, 52], the functional significance of these changes in vivo during *T. cruzi* infection remains unclear. Immune and inflammatory responses are not only mediated by cytokines produced by lymphocytes and other cell types but also by arachidonic acid metabolites (PGs and LTs). These cytokines and lipid mediators form a complex network with everyone and can regulate each other's production. Our findings raise the question that one mechanism for the NO production from plasma of infected 5-LO^−/−^ mice can be the increased production of IFN-*γ*. However, the mediators of this response still need to be identified.

Considering this complex net of cytokines and the influence of NOS system, evidence indicates that NO can modulate the biological levels of arachidonate-derived cell signaling molecules leading to either the stimulation or inhibition of its activity depending on the interaction with specific NO metabolites [53, 54]. COX metabolites are described as important mechanisms of host defenses against *T. cruzi* [55, 56].

PGE_2_ has the capacity of NO upregulation [57]; however, in our model, the mice did not reveal this correlation. Knockout mice displayed higher basal levels of prostaglandins in relation to the WT mice suggesting that lack of 5-LO could deviate the arachidonic acid metabolism to a further production of prostaglandins by cyclooxygenases. Infection did not alter the prostaglandin production in both groups, whereas the aminoguanidine and L-NAME treatments, associated or unassociated, led to a reduction in PGE_2_ levels from the 5-LO^−/−^ group.

In addition to NO, *T. cruzi* infection leads to the production of oxygen-reactive species induced by proinflammatory cytokines, as TNF-*α*, through mitochondrial free radical generation [58]. Additionally, NO is capable of reacting via multiple pathways to modulate lipid oxidation as well as the production of inflammatory and vasoactive eicosanoids by prostaglandin endoperoxide synthase and lipoxygenase [59].

Therefore, our results revealed that plasma lipoperoxidation is augmented during *T. cruzi* infection in both groups. iNOS and cNOS blockage presented distinct results: in the WT group, aminoguanidine treatment led to higher levels, whereas L-NAME reduced the oxidative stress to control levels. Moreover, in knockout mice, iNOS blockage reduced this event to the control levels, whereas cNOS presented a partial decrease in lipoperoxidation in relation to the untreated infected animals.

## 5. Conclusion

In summary, our study found that the NOS isoforms studied revealed opposite effects in response to acute infection with *T. cruzi*. cNOS appears to act via mechanisms that favor parasite survival, whereas iNOS modulates the infection by maximizing the host's trypanocidal mechanisms. Another important point is the regulation exerted by 5-LO metabolites over NOS activities and cytokine profile. It is important to conduct further studies in order to clarify the molecular mechanisms regulating host responses and allow an improved understanding of the NOS role in the complex net formed by cytokines and stress mediators during *T. cruzi* infection.

## Data Availability

The data used to support the findings of this study are available from the corresponding author upon request.

## Abbreviations

5-LO: 5-Lipoxygenase
5-LO^−/−^ mice: 5-Lipoxygenase knockout mice
WT: Wild type
iNOS/NOS_2_: Inducible nitric oxide synthase
cNOS: Constitutive nitric oxide synthase
NO: Nitric oxide
AG: Aminoguanidine
LN: L-NAME
LTs: Leukotrienes.
